# Closed-state inactivation involving an internal gate in Kv4.1 channels modulates pore blockade by intracellular quaternary ammonium ions

**DOI:** 10.1038/srep31131

**Published:** 2016-08-09

**Authors:** Jeffrey D. Fineberg, Tibor G. Szanto, Gyorgy Panyi, Manuel Covarrubias

**Affiliations:** 1 Department of Neuroscience, Vickie and Jack Farber Institute for Neuroscience at Thomas Jefferson University, Philadelphia, PA, USA; 2 Graduate Program in Molecular Physiology & Biophysics, Sidney Kimmel Medical College and College of Biomedical Sciences at Thomas Jefferson University, Philadelphia, PA 19107, USA; 3 Department of Biophysics & Cell Biology, Faculty of Medicine University of Debrecen, 4032 Debrecen, Hungary; 4 MTA-DE-NAP B Ion Channel Structure-Function Research Group, RCMM University of Debrecen, 4032 Debrecen, Hungary; 5 Vickie and Jack Farber Institute for Neuroscience at Thomas Jefferson University,Philadelphia, PA, USA.

## Abstract

Voltage-gated K^+^ (Kv) channel activation depends on interactions between voltage sensors and an intracellular activation gate that controls access to a central pore cavity. Here, we hypothesize that this gate is additionally responsible for closed-state inactivation (CSI) in Kv4.x channels. These Kv channels undergo CSI by a mechanism that is still poorly understood. To test the hypothesis, we deduced the state of the Kv4.1 channel intracellular gate by exploiting the trap-door paradigm of pore blockade by internally applied quaternary ammonium (QA) ions exhibiting slow blocking kinetics and high-affinity for a blocking site. We found that inactivation gating seemingly traps benzyl-tributylammonium (bTBuA) when it enters the central pore cavity in the open state. However, bTBuA fails to block inactivated Kv4.1 channels, suggesting gated access involving an internal gate. In contrast, bTBuA blockade of a Shaker Kv channel that undergoes open-state P/C-type inactivation exhibits fast onset and recovery inconsistent with bTBuA trapping. Furthermore, the inactivated Shaker Kv channel is readily blocked by bTBuA. We conclude that Kv4.1 closed-state inactivation modulates pore blockade by QA ions in a manner that depends on the state of the internal activation gate.

Voltage-gated K (Kv) channels are quintessential regulators of excitability in brain, heart and muscle. The voltage sensors of these ion channels respond to changes in membrane potential by undergoing rearrangements that in turn control the opening of the channel’s intracellular activation gate^1^,^2^,^3^. Significant evidence accumulated over the past two decades has confirmed the location of the main activation gate at the intracellular mouth of Kv channels^3^,^4^,^5^,^6^. Originally, Clay Armstrong used quaternary ammonium (QA) ions to deduce the intracellular location of a critical gate in squid Kv channels^7^,^8^. He demonstrated that quaternary ammonium (QA) ions occlude the pore at a deep site and are subsequently trapped in a pore cavity by the closing of an intracellular gate (trap-door paradigm). These findings stimulated the use of QA ions to probe the properties of pore cavities and gates in diverse K^+^ channels^9^,^10^,^11^,^12^,^13^,^14^. In particular, the Yellen lab applied the trap-door paradigm of QA ions to locate the intracellular activation gate in the *Drosophila* Shaker Kv channel^4^,^12^,^15^,^16^,^17^. Holmgren *et al*. predicted that, following open-channel blockade by the QA ion, hyperpolarization of the membrane potential would close an internal gate and trap the QA ion near its binding site in the pore. Accordingly, they found that depolarization following a hyperpolarization (after QA ion washout) induces ultra-slow current activation, indicating slow dissociation of the trapped QA ion and its escape from the pore. Therefore, Kv channels most likely have an intracellular activation gate that controls ion and pore blocker access to and escape from a central cavity^17^. Here, we hypothesize that an intracellular gate in Kv4.1 channels might play a novel role as inactivation gate when it fails to open.

An established classical mechanism of Kv channel inactivation identifies the cytoplasmic N-terminus of the channel as a tethered inactivation particle that occludes the open pore at an intracellular site (N-type inactivation)^18^,^19^. Another widely investigated classical mechanism involves a rearrangement in the selectivity filter, which acting as an external gate renders the pore non-conducting (P/C-type inactivation)^20^. N- and P/C-type inactivation are generally associated with open-state inactivation (to inactivate, the ion channel must open) of Shaker Kv channels. However, extensive evidence has shown that highly-conserved Kv4.1-3 channels with a full complement of accessory subunits (ternary complex including KChIP1 and DPP6; Methods) preferentially inactivate from a pre-open closed state (closed-state inactivation, CSI), which does not depend on the aforementioned classical mechanisms^21^,^22^,^23^.

Previously, Jerng *et al*. showed that the S6 segment double mutation of the PVPV bundle-crossing motif to PIPI in the activation gate of the Kv4.1 channel dramatically slows pore closing and inactivation^24^. In sharp contrast, the equivalent mutations in the Kv1.4 channel (a close mammalian relative of the *Drosophila* Shaker) only slowed pore closing without affecting inactivation^24^. These findings suggest that distinct interactions control classical inactivation and CSI in Kv1.4 and Kv4.1 channels, respectively^21^. Strengthening the implications of this assessment, the PVPM mutation in the Kv4.2 channel dramatically disrupts CSI and causes a severe neurological channelopathy^25^. Moreover, another study showed that critical inter-domain interactions generally responsible for pore opening in Kv channels might also control CSI gating in the Kv4.2 channel^26^. However, there is no conclusive evidence demonstrating that the intracellular activation gate could also be intimately involved in the mechanism of CSI, which is generally unknown^21^. A mechanism involving the intracellular activation gate is feasible if we assume that this gate can also adopt an alternate closed-inactivated conformation under depolarizing conditions that activate the voltage-sensing apparatus (Fig. 1). Here, we exploited the trap-door paradigm for QA ions to investigate a possible novel role of the intracellular activation gate acting as inactivation gate in the Kv4.1 channel with a full complement of accessory subunits^21^.

## Results

### P/C-type inactivation cannot trap a QA ion in the internal pore cavity of a Shaker Kv channel

**Figure 1:**
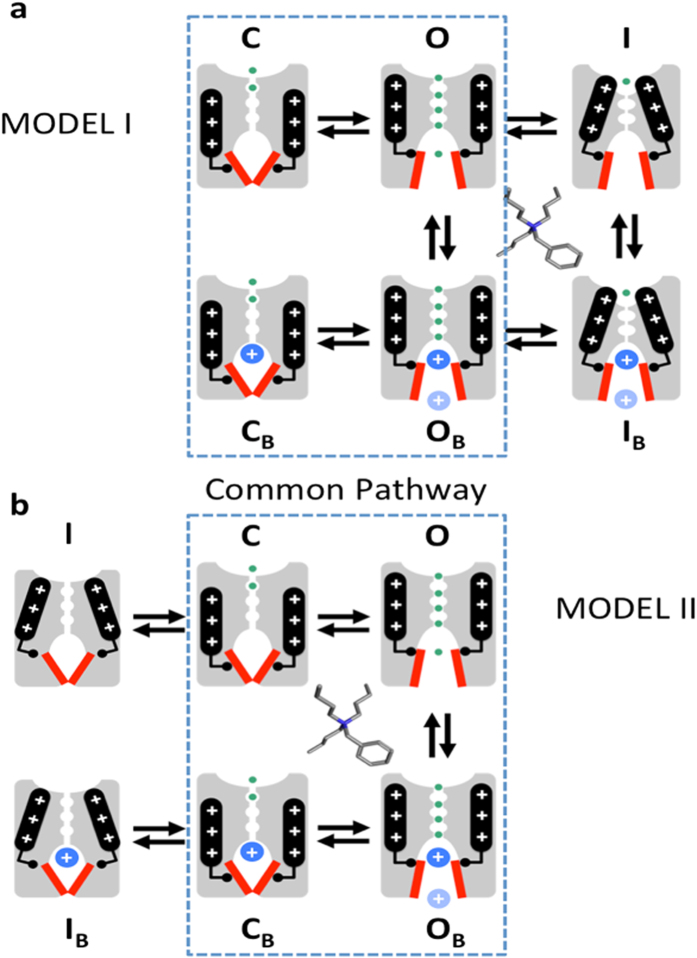
Working models: distinct trap-door scenarios in Shaker and Kv4.1 channels. (**a**) Cartoon representation of activation and P/C-type inactivation mechanisms in Shaker Kv channels (Model I). For clarity, only two opposing subunits of a Kv channel tetramer are represented in the cartoons. Top and bottom are the extracellular and intracellular sides of the channel, respectively. Voltage sensors (black rod with plus signs) undergo a voltage-dependent conformational change that controls the state of the intracellular activation gate (red bars). S4–S5 linkers (black elbows) interact with the activation gate to transmit the movement of the voltage sensors to the activation gate. Once this gate opens, the Kv channel allows transmembrane K^+^ flow (green circles) driven by an electrochemical potential. Closing of the activation gate traps the QA ion as shown by Holmgren *et al*.^4^. However, inactivation occurs at the external selectivity filter, which does not close the internal activation gate and, therefore, there is no QA ion trapping by inactivation. (**b**) Cartoon representation of a putative activation-inactivation mechanism in Kv4.1 channels (Model II). Note that closing of the internal gate traps the QA ion. Additionally, disconnecting the voltage sensors from the activation gate inactivates the channel as proposed by Shin *et al*.^37^ and maintains the trapped state. The dashed perimeters enclose common pathways in Models I and II.

To explore the possible role of the Kv4.1 channel activation gate in inactivation gating, we first used the trap-door paradigm to show that classical P/C-type inactivation does not involve closing of the internal activation gate (Fig. 1a, Model I). Previous studies have shown convincingly that the activation gate of Shaker Kv channels and certain prokaryotic K^+^ channels remains open as the selectivity filter adopts the non-conducting P/C-type conformation^27^,^28^,^29^,^30^,^31^,^32^. Thus, a Shaker Kv channel lacking N-type inactivation (Shaker-IR) but capable of undergoing P/C-type inactivation at the external selectivity filter might exhibit blockade by internally applied QA ions but would not be able to trap them by inactivation. Similarly, eukaryotic and prokaryotic Ca^++^-activated K^+^ channels control gating at the selectivity filter and do not exhibit gated access of QA ions to the pore’s central cavity^9^,^10^,^11^. To test the hypothesis, we used the mutant Shaker-IR T449K, which undergoes fast P/C-type inactivation developing with a time course similar to that of the ternary Kv4.1 channel complex^33^.

**Figure 2:**
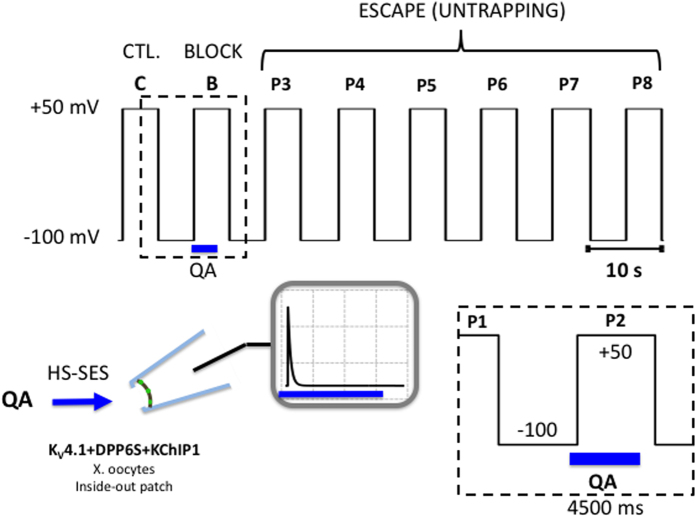
Voltage- and concentration-clamp protocol to probe for QA ion trapping by inactivation. Inside-out macropatches were subjected to eight 5-s depolarizing pulses (P1–P8, −100 to +50 mV) delivered at intervals of 5 s (10-s duty cycle). P1 (C) evokes the control current. P2 (B) evokes the current while the QA ion is applied to the internal side of the inside-out patch using a High Speed Solution Exchange System (HS-SES, Methods). The timing of the application in relation to P2 is shown in the boxed inset. Note that the exposure occurs while the channels undergo activation, opening and inactivation; and that this exposure is terminated 500 ms before the repolarization that closes the channels (once inactivation is complete and at steady-state). This duration is ~20-times longer than the exchange time of the solution switching system (Methods) and, therefore, it is unlikely that QA ions are still present in the intracellular bath solution at the time the channels close by repolarization. Following washout, P3–P8 are then used to test the recovery of the currents. P2, P3 and P4–P8 probe the block, trap and escape stages of the experiment, respectively. To confirm the reproducibility of the results, the P1–P8 sequence and QA ion application were generally repeated as many times as possible until the patch deteriorated.

We expressed the Shaker-IR T449K in *Xenopus* oocytes and applied a 5-s step depolarization from −100 to +50 mV to evoke the currents in the inside-out patch configuration, which is long enough to ensure complete steady-state inactivation (Methods; Fig. 2). This pulse (P1, Control, C) was terminated by a repolarization to −100 mV, allowing the channels to recover from inactivation at this membrane potential for 5 s before repeating the same pulse (Fig. 2). We began a 4.5-s internal concentration jump of 100 μM benzyl-tributylammonium (bTBuA) 500 ms before the next pulse (P2, Block, B) by means of a fast solution exchange system (Methods; Fig. 2). bTBuA is advantageous in these experiments because it is a slow open channel blocker with a long residency time that allows pore closing before dissociation^12^. The bTBuA application was quickly terminated by complete washout 500 ms before the end of P2 (~19 ms exchange time; Methods; Fig. 2). This sequence allowed for maximum exposure of the channels to bTBuA as they activated, opened and reached steady-state inactivation, while avoiding exposure to the QA ion as the channel closed by repolarization to −100 mV. This is critical because trapping might result from activation gate closing by repolarization. A previous study showed that closing by hyperpolarization in the presence of QA ions induces trapping in Shaker Kv channels^4^. After P2, we delivered six additional pulses (P3–P8) to ask whether pore blockade by trapping of bTBuA might have occurred behind the intracellular activation gate, and whether the current would eventually recover as bTBuA had the opportunity to escape every time a depolarizing voltage pulse opens the channels (Fig. 2).

**Figure 3:**
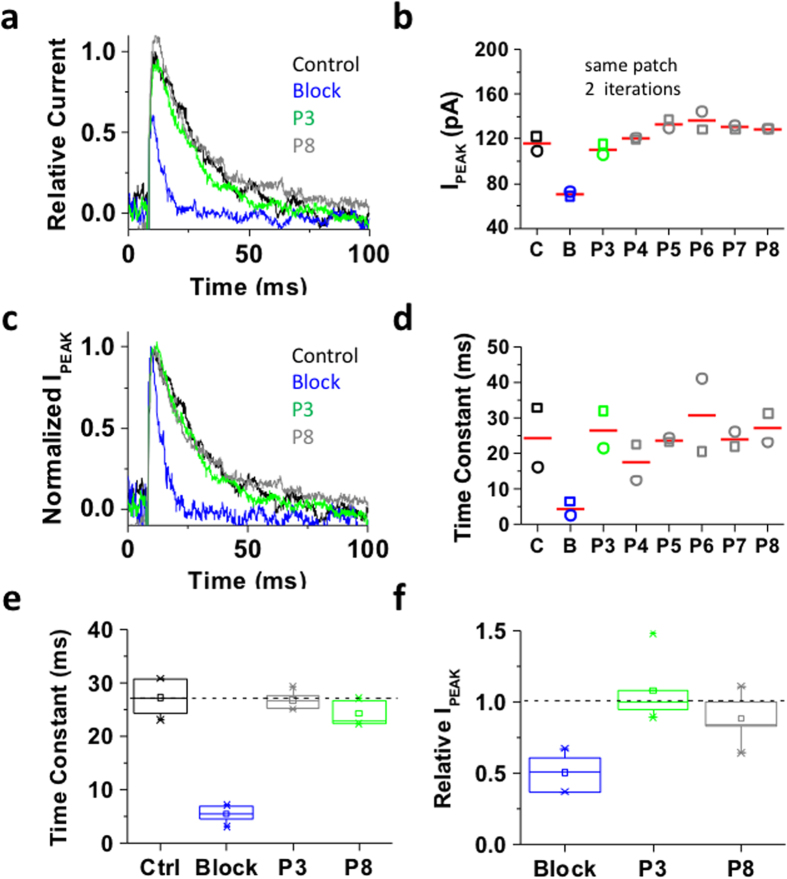
Inactivation of the Shaker-IR T449K channel cannot trap internally applied bTBuA. The mutant Shaker-IR T449K was heterologously expressed in *Xenopus* oocytes as explained under Methods. (**a**) Inside-out macropatch outward currents evoked by a step to +50 mV from a holding voltage of −100 mV. The overlaid traces depict the current profile before (black), during (blue) and after (green and gray) exposure of the intracellular side of the channel to 100 μM bTBuA. The exposure to the QA ion began before the step depolarization and was terminated after macroscopic inactivation reached steady-state (Fig. 2). Note that no exposure was allowed during the repolarizing step that closes the channels. (**b**) Magnitude of peak currents in two consecutive iterations of the experiment described above. Pulses P1 (Control, C), P2 (Block, B) and P3–P8 evoke the currents as explained in Fig. 2. (**c**) Scaled and normalized currents from panel (a). (**d**) Time constants of decay from the currents evoked by the test pulses as explained above for panel (a). Generally, one-two exponentials were sufficient to describe this decay. When the sum of two exponentials yielded the best fit, the reported time constant is the weighted average of the best-fit time constants. (**e**) Box plots of the time constants of current decay during blockade by bTBuA (blue box), after washout (green box) and from the currents evoked by P8 (N = 5 patches; 1–3 iterations each). (**f**) Box plots of the relative peak current amplitudes during blockade by bTBuA (blue box), after washout (green box) and from the currents evoked by P8 (N = 5 patches; 1–3 iterations each). On average, the peaks of currents evoked by P3 and P8 are not significantly different (Kruskal-Wallis, *p* = 0.37).

As expected, upon bTBuA application during P2 the peak current was reduced and current decay was accelerated, both typical features of QA-induced slow open channel block (Fig. 3a,b). This effect resembles the blockade of Shaker Kv channels by high-affinity QA ions^12^. Moreover, following QA ion washout, pulses P3–P8 evoked outward currents with magnitude and kinetics similar to that evoked by P1 (Fig. 3b–d), indicating complete recovery from pore blockade and no sign of cumulative inactivation resulting from slow recovery from inactivation (Fig. 3e,f). Thus, in contrast to closing by hyperpolarization, inactivation of Shaker-IR T449K does not appear to induce pore trapping of bTBuA. This result also shows that, following dissociation from the pore-blocking site, the QA ion exits the pore and is fully washed out before the next depolarizing pulse. We also expressed Shaker-IR T449K in *tsA-201* cells, which yielded larger currents (on-line Supplemental Information, Fig. 1S). In this system, 100 μM bTBuA quickly blocked most of the peak current, leaving only ~15% of control unblocked. However, resembling the results in *Xenopus* oocytes, blocker washout induced a quick current recovery that reached a plateau between P3 and P4 (80–85% of control; on-line Supplemental Information, Fig. 1S). Thus, bTBuA washout is sufficient to unblock most of the Shaker-IR T449K current. The incomplete recovery in *tsA-201* cells is in part due to 5–10% run-down or cumulative inactivation observed in the absence of blocker (on-line Supplemental Information, Figs 2S and 3S).

### Inactivation of the ternary Kv4.1 channel complex induces bTBuA trapping

**Figure 4:**
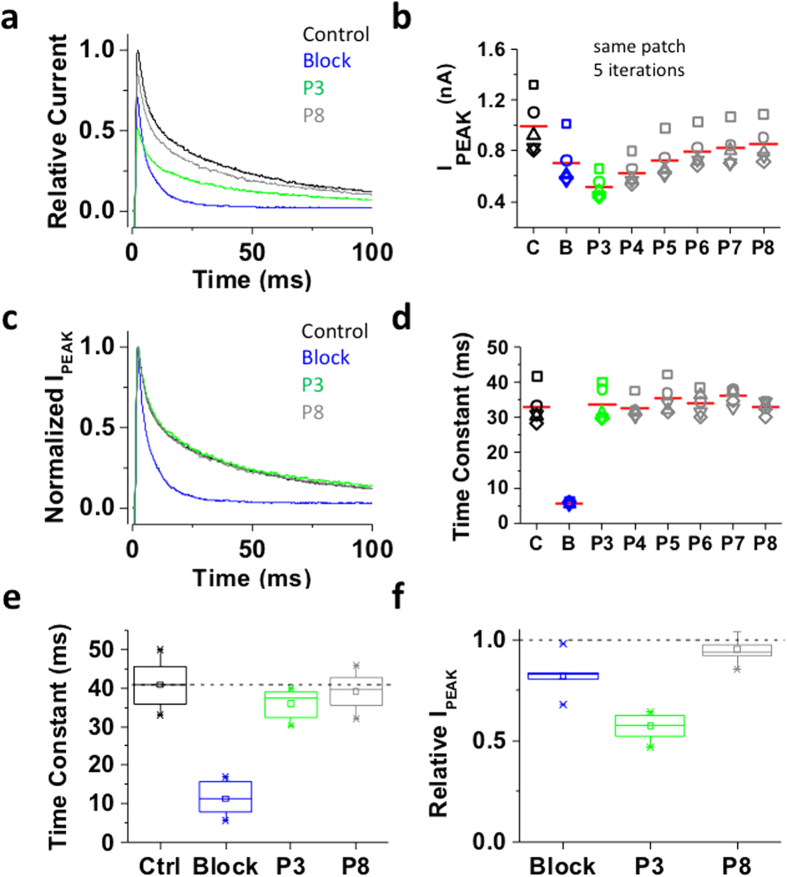
Inactivation of the ternary Kv4.1 channel complex traps internally applied bTBuA. The ternary Kv4.1 channel complex was heterologously expressed in *Xenopus* oocytes as explained under Methods. This complex includes the accessory subunits KChIP-1 and DPP6. (**a**) Inside-out macropatch outward currents evoked as described in Figs 2 and 3 legend. (**b**) Magnitude of peak currents in five consecutive iterations of the pulse protocol. Additional details as described in Fig. 3 legend. (**c**) Scaled and normalized currents from panel (a). (**d**) Time constants of decay from the currents evoked by the test pulses as explained in Fig. 3 legend. The reported time constant is the weighted average of the double exponential best-fit time constants. (**e**) Box plots of the time constants of current decay during blockade by bTBuA (blue box), after washout (green box) and from the currents evoked by P8 (N = 5 patches; 5–40 iterations each). (**f**) Box plots of the peak current amplitudes during blockade by bTBuA (blue box), after washout (green box) and from the currents evoked by P8 (N = 5 patches; 5–40 iterations each). On average, the peaks of currents evoked by P3 and P8 are significantly different (Kruskal-Wallis, *p* < 1.3 × 10^−11^).

**Figure 5:**
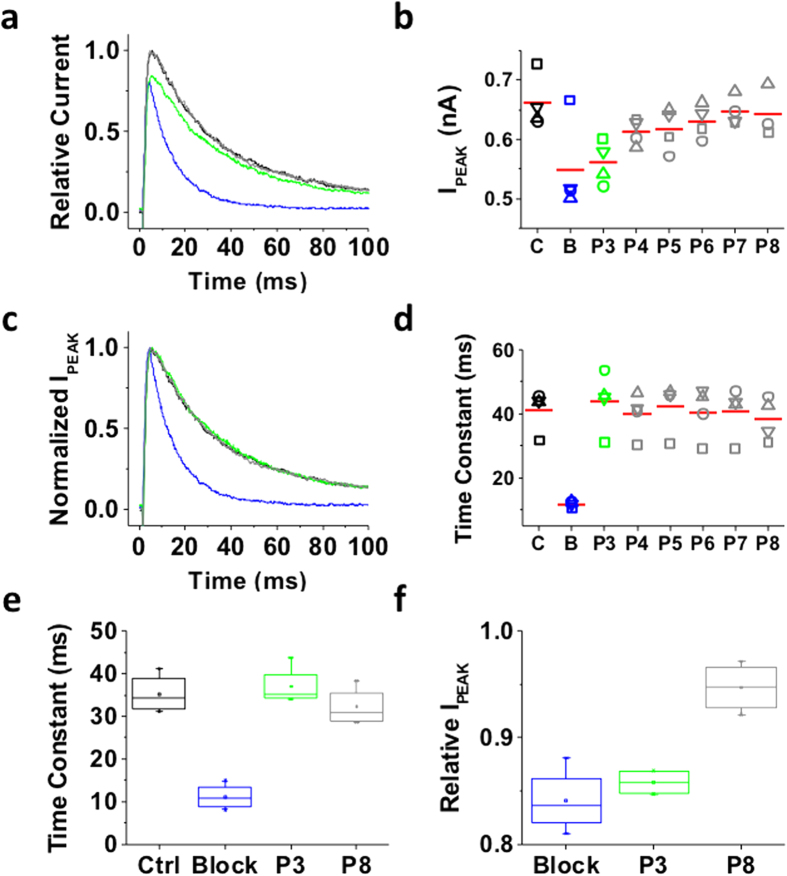
Lengthening the P2–P3 interval does not change block-trap-escape pattern produced by inactivation of the ternary Kv4.1 channel complex. (**a**) Inside-out macropatch outward currents evoked by a step to +50 mV from a holding voltage of −100 mV. The overlaid traces depict the current profile before (black), during (blue) and after (green and gray) exposure of the intracellular side of the channel to 100 μM bTBuA. The exposure to the QA ion began before the step depolarization and was terminated after macroscopic inactivation reached steady-state (Fig. 2). Note that no exposure was allowed during the repolarizing step that closes the channels. (**b**) Magnitude of peak currents in four consecutive iterations of the experiment described above. Pulses P1 (C, Control), P2 (B, Block) and P3–P8 evoke the currents as explained in Fig. 2. Critically, the interval between P2 and P3 was prolonged from 5 s (Fig. 4) to 40 s to eliminate the possibility of apparent QA ion trapping resulting from lack of recovery from inactivation at P3. (**c**) Scaled and normalized currents from panel (a). (**d**) Time constants of decay from the currents evoked by the test pulses as explained above for panel (a). (**e**) Box plots of the time constants of current decay during blockade by bTbuA (blue box), after washout (green box) and from the currents evoked by P8 (N = 5 patches; 4–12 iterations each). (**f**) Box plots of the relative peak current amplitudes during blockade by bTbuA (blue box), after washout (green box) and from the currents evoked by P8 (N = 5 patches; 4–12 iterations each). On average, the peaks of currents evoked by P3 and P8 are significantly different (Kruskal-Wallis, *p* < 0.001).

**Figure 6:**
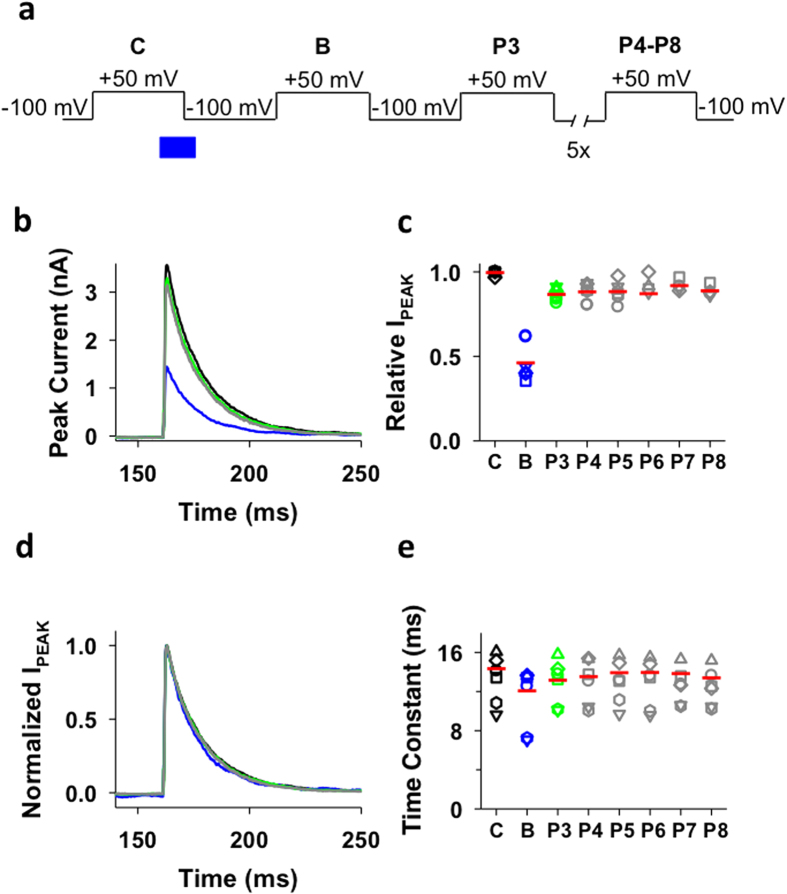
Inactivated Shaker–IR T449K allows the access of bTBuA to the pore and the blocker gets trapped in the pore upon hyperpolarization. T449K channels were transiently expressed in *ts-A201* cells (see Methods). (**a**) Voltage- and concentration-clamp protocol to probe for bTBuA ion trapping by inactivation. (**b**) Macroscopic outward currents were recorded in inside-out patches. The patches were repeatedly depolarized from a holding potential of −100 to +50 mV for 5 s to ensure complete inactivation. The nomenclature of the pulses in the sequence is in panel a. The interpulse interval was 5 s and the patches were held at the holding potential between the pulses. The intracellular side of the patch was exposed to 100 μM bTBuA for 1.5 s (1 s at +50 mV and 500 ms at −100 mV). The timing and the duration of the bTBuA pulse are indicated by the *solid* blue bar. The overlaid traces depict the currents recorded in control solution (P1, C=control, black), during the first pulse following the exposure of the intracellular side of inactivated channels to 100 μM bTBuA (P2, B=block, blue) and during subsequent pulses in control solution (P3, green, and P4–P8, grey) (only 100-ms-long segment of the currents is shown for clarity). (**c**) Scatter plots of the normalized peak current amplitudes measured during pulses C, B, and P3–P8 (see above). Horizontal bars indicate the mean of N experiments. (**d**) Currents in panel b were normalized to their respective peak and shown as a function of time. Color code is the same as in panel a. (**e**) Scatter plot of the individual time constants obtained from currents evoked by pulses C, B, and P3–P8 (see above) and the mean of the time constants (horizontal bars). Inactivation time constant of the current at +50 mV was determined by fitting a single exponential function to the decaying part of the currents.

**Figure 7:**
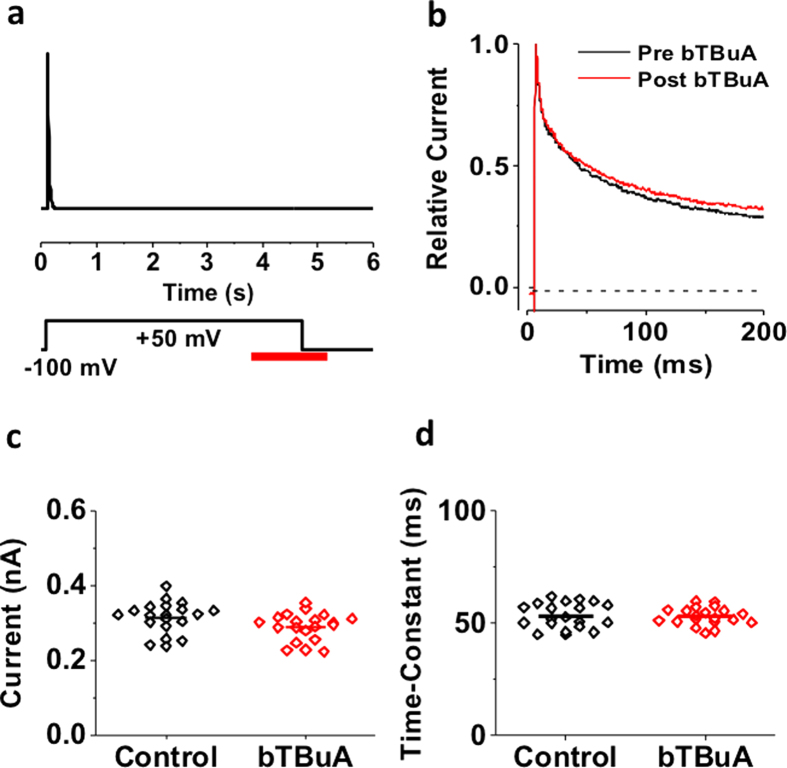
Inactivation of the ternary Kv4.1 channel complex prevents internal access of bTBuA to the pore. (**a**) Schematic of experimental protocol. Current was evoked by a 5-s step depolarization from −100 to +50 mV to ensure complete steady-state inactivation. The intracellular side of the patch was exposed to 100 μM intracellular bTBuA for a total of 1.5 s (red bar). The application began once current inactivation had reached steady-state, and continued for 1 s at +50 mV. The exposure to bTBuA was then terminated 500 ms after repolarizing the membrane to −100 mV. To test whether bTBuA had any effect, the same pulse was subsequently repeated while the inside patch was bathed in control intracellular solution. (**b**) Overlay of macropatch currents before (black) and after (red) intracellular exposure to bTBuA. (**c**) Comparison of peak currents before (control) and after (bTBuA) exposure (20 iterations of the pulse sequence on the same macropatch). (**d**) Comparison of weighted time constant of decay from currents before (control) and after (bTBuA) exposure (same experiment, 20 iterations). Horizontal lines in (**c**,**d**) indicate mean values.

In contrast to the Shaker-IR T449K channel that undergoes open P/C-type inactivation at an external site involving the selectivity filter, the ternary Kv4.1 channel complex undergoes preferential CSI possibly involving the internal activation gate (Fig. 1b, Model II). Would the state of this gate hinder/modulate the interaction with an internally-applied QA ion? To answer this question, we applied the experimental protocol tested on Shaker-IR T449K to the ternary Kv4.1 channel complex (Fig. 2). Under control conditions, P1 induced a fast activating and inactivating outward current characteristic of the ternary Kv4.1 channel complex (Fig. 4a). In the inside-out patch configuration, Kv4.1 current decay kinetics may exhibit acceleration and apparent heterogeneity induced by modulation of CSI (compare current decays in Figs 4, 5, 6, 7)^34^. To probe the conformation of an internal gate once inactivation was fully developed, we extended the exposure to bTBuA for several seconds after the current had decayed to a steady-state level (Fig. 2). In the presence of 100 μM bTBuA, P2 evoked an outward transient current with reduced peak amplitude and accelerated decay, demonstrating again that the QA ion induced slow open channel block (Fig. 4a). Following washout, however, the peak current evoked by P3 remained reduced (Fig. 4a,b), whereas current decay slowed back to the control level (Fig. 4c,d). These results show that current inhibition was not immediately reversible upon complete washout (~19 ms exchange time; Methods), suggesting sustained blockade possibly resulting from pore trapping of bTBuA or very slow dissociation of the blocker. The current evoked by subsequent voltage pulses (P4–P8) gradually returned to its original amplitude, as bTBuA dissociates and escapes from the pore cavity every time the intracellular activation gate opens (Fig. 4b). The sustained inhibition after bTBuA removal was not due to lack of recovery from inactivation because we observed the same sustained blockade and slow unblocking when the P2–P3 inter-pulse interval was 13-times longer (40 s; Fig. 5a,b). Also, indicating that normal unblocked channels underlie the currents evoked by P3–P8, the decay kinetics of the currents are similar to those from the control response (Figs 4c,d and 5c,d). Tetrabutylammonium (TBA) additionally yielded results qualitatively similar to those described above, demonstrating that inactivation of the ternary Kv4.1 channel complex can also trap another QA ion capable of inducing slow pore blockade^4^. Despite variable kinetics of current decay (above) and independently of inter-pulse interval length (3 vs. 40 s), we observed similar patterns when comparing results from multiple patches (Figs 4e,f and 5e,f).

### Differential roles of the internal activation gate in Shaker-IR T449K and Kv4.1 channels

The contrasting results obtained with Shaker-IR T449K and the ternary Kv4.1 channel complex could have resulted from an intrinsic inability of the former to trap bTBuA in the pore cavity. To examine this possibility and additionally establish that the internal activation gate of Shaker-IR T449K is open in the inactivated state, we exposed this channel to internal bTBuA once inactivation was complete and probed its effect following closing of the gate by hyperpolarization in the presence of the blocker (Fig. 6a). In this case, blockade would occur silently because the channels are inactivated at the onset of blocker application; however, if bTBuA had blocked the pore by entering the cavity and dissociates slowly (relative to gating), closing of the activation gate by hyperpolarization might trap bTBuA. Following complete washout, we found that the peak current evoked by the next pulse was ~50% of control, demonstrating that bTBuA had entered the pore of open-inactivated channels to block them (Fig. 6b,c). Blockade is now observable because hyperpolarization-induced closure of the activation gate most likely trapped the QA ion in the pore cavity. Ruling out re-blocking by residual bTBuA, current kinetics remained unaffected, which is to be expected if the remaining current simply represents unblocked channels (Fig. 6d,e). Exit of the blocker was, however, relatively quick. The peak current evoked by P3 was ~90% of control and remained at that level until the end of the experiment (Fig. 6c). Furthermore, bTBuA could only enter the pore of the open-inactivated channels because internal application of this QA ion in the closed state only resulted in no detectable blockade (on-line Supplemental Information, Fig. 3S). These results suggest that bTBuA can access the pore of the open-inactivated Shaker-IR T449K, which is capable of QA ion trapping upon closure of its activation gate by hyperpolarization. Silent blockade in the open-inactivated state was at steady-state because extending the bTBuA exposure at +50 mV did not decrease current further (on-line Supplemental Information, Fig. 4S). Also, we observed no further blockade by extending the exposure to bTBuA at −100 mV because hyperpolarization-induced gate closure controls blockade by trapping (on-line Supplemental Information, Fig. 4S).

If the internal activation gate of inactivated Kv4.1 channels is capable of acting as a trap-door to physically keep QA ions in the pore (Fig. 1b, Model II; Figs 4 and 5), it should also control access of the QA ion to its pore blocking site (gated access hypothesis). To test this hypothesis, we used the application protocol previously tested on Shaker-IR T449K (Fig. 6). Kv4.1 channels were exposed to 100 μM intracellular bTBuA for 1 s once inactivation had reached steady-state at +50 mV, and terminated this application 500 ms after repolarizing the membrane to −100 mV (Fig. 7a). Even at +50 mV, preferential CSI eventually occurs anytime channels sojourn back into the inactivation-permissive preopen closed state presumably controlled by the intracellular activation gate (Fig. 1b, left arm of pathway). However, as it is in Shaker-IR T449K (Fig. 6), if inactivation occurs at a gate external to the pore cavity and the intracellular activation gate is almost always open at +50 mV, bTBuA would access its binding site in the pore cavity even though channels are inactivated. QA ion trapping could then only occur upon closure of the intracellular activation gate by a strong repolarization. Contrary to this prediction, we found that a test pulse delivered after blocker exposure, repolarization and washout (as described above) evoked currents that were indistinguishable from the control currents recorded in the absence of bTBuA, in terms of both magnitude and kinetics (Fig. 7b). Multiple trials of the pulse sequence on the same patch induced little to no change in current amplitude and decay kinetics (Fig. 7c,d). In three independent experiments (N = 3 patches), the control peak currents and those measured after exposure to bTBuA and washout were: 1.1 and 1.1 nA, N = 20 trials; 0.39 and 0.36 nA, N = 30 trials; 0.99 and 0.96 nA, N = 30 trials. The mean ± SD peak change was −4.1 ± 4.4%. This result differs sharply from the behavior of Shaker-IR T449K channels, which are blocked by bTBuA in the open-inactivated state and seemingly trap the QA ion by hyperpolarization-induced closure of the activation gate (Fig. 6). This is also in contrast to the sustained blockade observed when the QA ion enters the pore of conducting Kv4.1 open channels and becomes presumably trapped by inactivation (Figs 4 and 5). Therefore, the activation gate of the ternary Kv4.1 channel might play a distinct role by adopting a closed conformation in the inactivated state, which controls pore access and exit of QA ions. By contrast, the activation gate of Shaker-IR T449K is open in the inactivated state and can only control pore access and exit of QA ions by deactivation and closing.

### Inactivation of the ternary Kv4.1 channel complex cannot trap TEA

**Figure 8:**
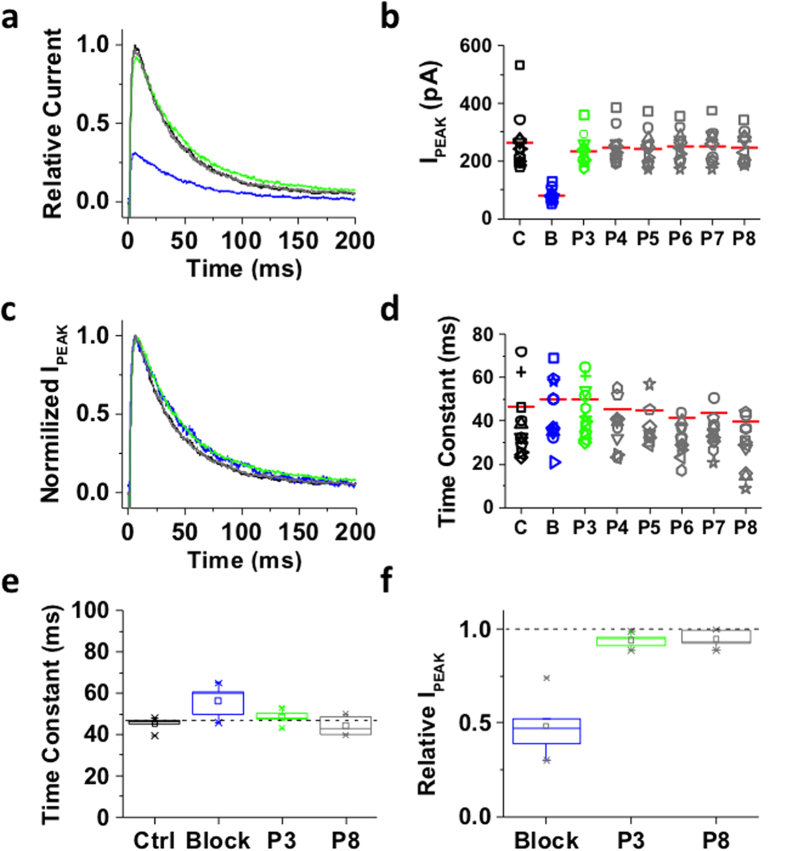
Inactivation of the ternary Kv4.1 channel complex cannot trap internally applied TEA. The ternary Kv4.1 channel complex was heterologously expressed in *Xenopus* oocytes as explained under Methods. (**a**) Inside-out macropatch outward currents evoked as described in Figs 2 and 3 legend. (**b**) Magnitude of peak currents in eleven consecutive iterations of the pulse protocol. Additional details as described in Figs 2 and 3 legend. (**c**) Scaled and normalized currents from panel a. (**d**) Time constants of decay from the currents evoked by the test pulses as explained in Fig. 3 legend. (**e**) Box plots of the time constants of current decay during blockade by TEA (blue box), after washout (green box) and from the currents evoked by P8 (N = 5 patches; 5–25 iterations each). (**f**) Box plots of the peak current amplitudes during blockade by TEA (blue box), after washout (green box) and from the currents evoked by P8 (N = 5 patches; 5–25 iterations each). On average, the peaks of currents evoked by P3 and P8 are not significantly different (Kruskal-Wallis, *p* = 1).

Reflecting high binding affinity, intracellularly applied bTBuA behaves as slow open channel blockers with relatively long residency times in their blocking sites^7^,^14^. Therefore, as shown by others, rapid closing of the Kv channel intracellular activation gate by repolarization/hyperpolarization would readily trap these QA ions following open channel blockade^4^. TEA, by contrast, is a fast intracellular open channel blocker with rapid binding/unbinding kinetics, which renders pore trapping by closing of the intracellular activation gate more difficult. In light of these properties, we predicted that, in contrast to the results with bTBuA and TBA, ternary Kv4.1 channels would not be able to display apparent trapping of TEA by inactivation. To test this hypothesis, we carried out the TEA experiment under conditions identical to those used to test bTBuA (Figs 2 and 8). Consistent with rapid blockade kinetics by 10 mM intracellular TEA, the current was inhibited by ~50% without affecting the kinetics of current decay (Fig. 8a–c). Moreover, following complete removal of TEA in the bath solution, P3–P8 evoked outward currents exhibiting control amplitude and kinetics (Fig. 8b–d). Such a rapid recovery indicates that no TEA remained in the pore as the current reached its peak in response to P3. Therefore, the low-affinity blocker TEA exhibits fast blocking kinetics but no evidence of a delayed escape otherwise expected from a high-affinity QA ion with a long residency in pore that favors trapping by an internal gate.

## Discussion

QA ions capable of blocking K^+^ channels from the intracellular side have been used extensively to characterize their gates^4^,^7^,^8^,^9^,^10^,^11^,^12^,^13^,^14^,^15^,^16^. These works along with cysteine accessibility and structural studies have produced compelling evidence for an intracellular gate that controls opening of K^+^ channels^2^,^5^,^6^,^35^. In Kv channels, the narrowest part of the S6 bundle crossing most likely constitutes this gate, which communicates with the S4 voltage sensors via the S4–S5 linkers from individual subunits to control voltage-dependent activation^2^. This framework helped us design experiments to deduce the states of an internal gate following inactivation. The following key observations are consistent with an operational block-trap-escape model in which inactivation might result from the failure to open an intracellular activation gate (Fig. 1b, Model II; Figs 4, 5, 6, 7, 8). First, as expected for an internally-applied slow open channel blocker with high-affinity, the QA ion bTBuA reduces the peak current and accelerates current decay. This decay may reflect the kinetics of slow open channel block (see below). Second, following complete inactivation and blocker removal, the peak current does not immediately recover to its control level, albeit the decay kinetics fully return to the control profile within the next pulse (P3). These observations suggest that the QA ion occupied a deep internal site within the K^+^ channel pore and that closing of a gate at the intracellular pore entrance might have trapped the QA ion in the pore. Third, QA ions can escape upon repetitive stimulation, such that every time the channel recovers from inactivation, and the intracellular activation gate opens again in response to a subsequent depolarization, there is opportunity for the blocking QA ion to leave the pore. As the channels become unblocked, the outward current progressively recovers to its control level. The progressive current recovery is not likely to reflect a QA ion-induced slow recovery from inactivation because prolonging the interval between P2 and P3 from 3 to 40 s was not sufficient to return the outward current to its control level at P3. Unblocking induced by repetitive stimulation was necessary and sufficient to recover the control level of the outward current. Additionally consistent with the idea of an internal gate that fails to open when Kv4.1 channels inactivate, bTBuA cannot access the internal pore cavity and its binding site once inactivation is fully developed and complete. Gated access strongly suggests that a closed internal gate in the inactivated state protects the binding site for QA ions in the pore.

Two additional independent results further support a distinct block-trap-escape sequence induced by inactivation in Kv4.1 channels. First, under otherwise identical conditions, the Shaker-IR T449K channel exhibited slow blockade by bTBuA, which was then readily relieved by washout because the internal activation gate of this channel remains open during P/C-type inactivation at the external selectivity filter^29^. We validated this result by 1) qualitatively recapitulating the aforementioned Shaker-IR T449K channel behavior in *tsA-201* cells, and 2) showing that bTBuA can access and block the pore of this channel in the open-inactivated state. This result is particularly significant because it shows that the activation gates of the Shaker Kv channel and the ternary Kv4.1 channel complex adopt distinct conformations in their corresponding inactivated states (open and closed, respectively).

Second, TEA, which is an open channel blocker with fast blocking/unblocking kinetics, effectively blocked the Kv4.1 current but exhibited no apparent trapping by inactivation. TEA’s residency time is not long enough to allow observable trapping. Since the bulk bath concentration of TEA dropped to zero following its removal, rapid dissociation and escape occurs as soon as the pore opens again. The stickier bTBuA, by contrast, experiences a different fate resulting from its longer residency at the blocking site in the pore and eventual trapping in the central pore cavity as the internal gate closes behind it and fails to re-open (Fig. 1b, Model II).

Previous studies of Shaker Kv channels reported that internally applied QA ions interact with distinct pore sites and influence the rate of P/C-type inactivation in two ways^15^,^16^. Pore blockade by high-affinity QA ions (e.g., long-chain alkylammonium derivatives) stops K^+^ flux and thereby starves a K^+^ binding site at the external mouth of the selectivity filter, which accelerates the conformational change underlying P/C-type inactivation and induces use-dependent inactivation. Independently of K^+^ pore occupancy, TEA ions may alternatively slow P/C-type inactivation allosterically through distinct interactions. For both Shaker Kv and Kv4.1 channels under normal ionic conditions, we have interpreted the accelerated current decay induced by bTBuA as a reflection of pore blockade kinetics. This interpretation is generally consistent with the slow open pore blockade mechanism associated with high-affinity long-chain alkylammonium derivatives^12^. Currently, it is not clear whether the aforementioned effects of QA ions on P/C-type inactivation also contribute to the net rate of current decay in our experiments. Notably, however, while TBA and bTBuA reduce the peak Kv4.1 current and induce accelerated current decay, TEA only reduces the peak current with no effect on current kinetics. These features are expected for the fast low-affinity TEA blockade and the slow high-affinity blockade induced by the bulkier and more hydrophobic QA ions, such as TBA and bTBuA^12^. Despite the specifics of the blocking mechanisms, there are sharp differences with respect to the off-pathway of blockade when comparing the Shaker Kv channel to the Kv4.1 channel, as discussed above. In the inactivated Kv4.1 channel, the closing of an internal gate might restrict bTBuA pore entry and exit (Figs 4,5 and 7). By contrast, bTBuA blockade is readily relieved upon washout in the P/C-type inactivated Shaker Kv channel (Fig. 3). This is reminiscent of the inability of big conductance Ca^2+^-gated K^+^ (BK) channels to trap QA ions upon deactivation^10^. Thus, a gate at the intracellular mouth of the channel is not controlling opening of the pore in BK channels. Rather, as shown for Ca^2+^–dependent MthK channels^11^, a gate external to the pore’s central cavity (possibly the selectivity filter itself) is the actual activation gate of these channels.

Instead of physical trapping by an internal gate, Kv4.1 inactivation might induce ultraslow blocker dissociation from its binding site in the pore (i.e., enhancing binding affinity in a state-dependent manner). In such scenario, QA trapping could have resulted from classical repolarization-induced closing of the activation gate rather than from inactivation developing during the depolarizing step (bTBuA might still be in the pore by the time the membrane potential is repolarized 500 ms after washout). P/C-type inactivation in Shaker Kv channels causes rearrangements in the pore cavity, which greatly reduce TEA affinity^29^. However, Kv4.1 and Shaker Kv channels with highly homologous pore lining regions (selectivity filter and S6 segments are 70% identical) yielded radically different results (Figs 3, 4, 5, 6, 7, 8), and the Kv4.1 channel does not undergo P/C-type inactivation^21^. Nevertheless, a state-dependent increase in bTBuA affinity (rather than trapping by an internal gate) as an alternative explanation for the results (Figs 4 and 5) cannot be ruled out; however, such an alternate scenario would additionally require assuming that a distinct mechanism of CSI in Kv4.1 channels has effects on QA binding that are opposite to those induced by P/C-type inactivation in Shaker Kv channels. Rather than an increase in blocker affinity, inactivation-dependent gated access (Fig. 7) is independently consistent with the physical trapping interpretation of the results from Kv4.1 channels (Fig. 1b, Model II). Whether apparent gated access induced by inactivation results from a sharp state-dependent decrease in bTBuA affinity (contrary to the argument above) would also require assuming that a non-P/C-type inactivation mechanism of CSI in Kv4.1 channels (which might not involve the activation gate) allosterically modulates QA binding affinity. More work that includes analysis of QA ion binding sites will be necessary to test the complex state-dependent scenarios outlined above. For instance, the key QA ion-binding residue T441 (at the internal mouth of the selectivity filter) is conserved in Kv4.1 and Shaker channels^36^; however, T469, another key residue for QA ion-binding in Shaker is replaced with valine in Kv4.1^15^.

In light of the distinct ability of Kv4.1 channel CSI to modulate pore entry and exit of bTBuA, we propose a novel mechanism of Kv channel CSI involving an intracellular inactivation gate that controls access to the central cavity of the channel. Since this ability resembles a characteristic property of the activation gate in Shaker Kv channels, it is parsimoniously possible that this gate plays a dual role controlling activation and inactivation in highly conserved Kv4.x channels (Fig. 1). To visualize this idea, we drew inspiration from the “slippage” mechanism of inactivation proposed for HCN channels by Yellen *et al*.^21^,^37^. Generally, the S4–S5 linkers in the Kv channel tetramer transmit the movement of the S4 voltage sensor and must experience a strong concerted interaction with the activation gate (the intracellular S6 tails adjacent to the bundle crossing) to quickly open the pore (Fig. 1, C to O transition)^2^. However, if the interaction between the S4–S5 linkers and the intracellular tail of the S6 segments in the pre-open closed state is weak, pore opening might fail (Fig. 1b, Model II; C to I_C_ transition). Essentially, disengaged voltage sensors lose the ability to control pore opening rendering the Kv channel “desensitized” to voltage^37^,^38^. Failing to open in this novel mechanism is thus directly responsible for inactivation, which leaves the activation gate (the S6 bundle crossing) in an alternate closed-inactivated conformation (Fig. 1b, Model II, I_C_). One can then see that every time the pore containing a QA ion closes as the channel re-enters the pre-open closed state (Fig. 1b, O_B_ to C_B_ transition), the activation gate might adopt the closed-inactivated conformation effectively trapping the QA ion in the central cavity (Fig. 1b, C_B_ to I_B_ transition). QA ion escape may only occur every time pore opening actually succeeds. Note that inactivation-induced QA ion trapping cannot occur in a typical Shaker Kv channel, where the strong interaction between the S4–S5 linker the S6 tails effectively controls pore opening, and inactivation occurs at the external selectivity filter gate (Fig. 1a, Model I). Although the proposed model provides a compelling economical explanation for the results, further work is necessary to confirm that inactivation gating of highly conserved Kv4.x channels directly results from the failure to open the intracellular activation gate. In particular, because C_B_ and I_B_ are electrically silent, it would be important to determine whether QA ion binding only stabilizes the pre-open closed conformation in the CSI pathway or remains bound and possibly trapped in the CSI conformation.

## Methods

### Molecular Biology and Heterologous Expression in *Xenopus* oocytes

The cDNAs were obtained from the following sources: rDPP6-S (rat dipeptidylpeptidase-like protein 6, short variant), B. Rudy (New York University, New York, NY); Human KChIP-1 was maintained in a modified pBluescript vector, pBJ/KSM (gift from M. Bowlby, Wyeth-Ayerst Research, Princeton, NJ); mKv4.1 (mouse orthologue), M. Covarrubias (Thomas Jefferson University, Philadelphia, PA); *Drosophila Shaker* B, T. Hoshi (University of Pennsylvania, Philadelphia, PA). These cDNAs were maintained in *E. coli*. and purified for further work using commercially available kits (Qiagen). Site-directed mutagenesis was carried out using the Quick Change Mutagenesis kit as indicated by the manufacturer (Stratagene). Mutations were confirmed by automated DNA sequencing at the Cancer Genomics Shared Resource of the Kimmel Cancer Center. The mRNA for heterologous expression was produced by *in vitro* transcription using the mMessage mMachine kit driven by the T3 or T7 RNA polymerase (Ambion) and purified using the RNeasy kit (Qiagen). For heterologous expression and electrophysiological recording, *Xenopus laevis* oocytes were harvested and microinjected as described previously^34^,^39^. All animal experiments were approved by the Institutional Animal Care and Use Committee (IACUC) of Thomas Jefferson University and were conducted in accordance with its guidelines and regulations. To express the ternary Kv4.1 channel including pore-forming and auxiliary subunits, we injected mRNAs in the proportions previously described by Wang *et al*.^40^. Oocytes were kept at 18 °C in Leibovitz’s solution (Gibco) with 45% H_2_O by volume, 0.24% HEPES and 0.1% Gentamicin, pH 7.40. Experiments were generally carried out 1–3 days post mRNA injection.

### Mammalian Cell Culture and Transfections

The *tsA-201* cells, originally obtained from Dr. Richard Horn (Thomas Jefferson University, Philadelphia, PA), are conventional human embryonic kidney cells (HEK-293 cells) transformed with SV40 large T antigen (Sigma-Aldrich). These cells were grown in DMEM-high glucose supplemented with 10% FBS, 2 mM l-glutamine, 100 U/ml penicillin-g, and 100 μg/ml streptomycin (Invitrogen) at 37 °C in a 9% CO_2_ and 95% air humidified atmosphere. Cells were passaged twice per week following a 7-min incubation in Versene containing 0.2 g EDTA/L (Invitrogen). A Calcium Phosphate Transfection Kit (Invitrogen) was used to co-transfect a GFP plasmid vector along with the Shaker-IR construct. Transfected cells were re-plated onto 35-mm polystyrene cell culture dishes pretreated with Poly-L-Ornithine (Sigma-Aldrich) to improve cell adhesion for excising patches. Channels were transiently expressed in *tsA201* cells 12–48 hours after transfection. The GFP positive cells were identified by a Nikon TS-100 fluorescence microscope using bandpass filters of 455–495 nm and 515–555 nm for excitation and emission, respectively. Experiments in Fig. 6, S1–S4 were conducted with *tsA-201* cells.

### *Xenopus* Oocyte Electrophysiology

Patch-clamp experiments in *Xenopus* oocytes were carried out in the inside-out patch configuration^34^. Internal (bath) solution contained (mM): 98 KCl, 0.5 MgCl_2,_ 1 EGTA and 10 HEPES, pH 7.2. External (pipette) solution contained (mM): 96 NaCl, 2.5 Na-Pyruvate, 2 KCl, 1.8 CaCl_2_, 1 MgCl_2_ and 5 HEPES, pH 7.4. Patch-clamp experiments were performed using patch electrodes made of thin-walled 8520 Custom glass (Warner Instruments) pulled with a PIP5 micropipette puller (HEKA Instruments Inc.). Patch electrodes were coated with Sylgard elastomer (Dow Corning) and had tip resistances of 1–3 MΩ. Currents were low-pass filtered at 2 kHz (Axopatch 200B internal four-pole Bessel filter) and sampled at 10 kHz using the Digidata 1322A and Clampex 9.2 (Molecular Devices). All patch-clamp recordings were conducted at room temperature (22–24 °C).

### Determination of solution switching and exchange times

A High-Speed Solution Exchange System (HS-SES, ALA Scientific) was used for rapid exposure control of inside-out patches to intracelluarly applied QA ion derivatives. The solution switching time of the system was determined by measuring the current resulting from the change in the liquid junction potential of the open patch electrode when switching from 150 mM NaCl in the pipette and the bath to 75 mM KCl (and 75 mM NaCl) in the bath. The switching time was measured as the elapsed time from 10% to 90% of the total change in current. Typically, 50 iterations of the concentration-clamp protocol applied to the same patch were acquired and averaged to determine the switching time. For N = 4 patches, the mean switching-times were 2.9 ± 0.4 ms and 3.9 ± 2.1 ms, for the on and off phases of the concentration-jump, respectively.

The inside-out patch solution exchange time was determined by using Shaker IR/T449V channels expressed in *Xenopus* oocytes. These mutant channels exhibit little to no inactivation. Inside-out patches were exposed to the normal bath solution and a 5-second step depolarization from −100 to +50 mV. Then, 1.5 s after the onset of the voltage pulse, the bath solution was switched to 49 mM KCl (and 49 mM NaCl). The concentration jump typically lasted 2 s. The resultant change in electrochemical driving-force for K^+^ yielded a change in current amplitude, and the exchange time was measured as the elapsed time from 10% to 90% of the current change. For a given inside-out patch, this combination of voltage- and concentration-clamping was repeated 50 times. The average exchange times from N = 4 patches were 19.8 ± 1.6 ms and 18.3 ± 0.9 ms for the on and off phases of the concentration jump, respectively. Throughout the course of this study, the exchange times ranged between 20–100 ms, depending on patch geometry. The QA concentration-jump experiments spanned 10 times longer on-off durations than the slowest exchange time.

### Mammalian Cell Electrophysiology

All ionic current experiments were performed using excised inside-out patches. Typical current amplitudes were 300–2000 pA at +50 mV test potential, allowing the recording of macroscopic currents. Only those cells with a steady-state current <5% of the peak current were used in the experiments. All measurements were carried out by using Axopatch 200B amplifier connected to a personal computer using Axon Digidata 1550A data acquisition hardware, respectively (Molecular Devices). In general, the holding potential was −100 mV. Experiments were done at room temperature ranging between 20–24 °C. Data were analyzed using the pClamp 10 software package (Molecular Devices). Before analysis, current traces were digitally filtered with a three-point boxcar smoothing filter. A Warner Instruments SF-77A Perfusion Fast-Step system with three-barrel square glass (700 μm internal diameter) was used for rapid solution exchange. This system had a 10–90% exchange time for inside-out patches between 20–30 ms.

### Data Analysis

Graphical display and empirical curve fitting were conducted in Clampfit 10.2 (Molecular Devices) and OriginPro 8.0 (OriginLab Corp.). Current decay (inactivation) kinetics were described empirically by an exponential or sum of exponential terms. Patch excision accelerates Kv4.1 inactivation^34^, which sometimes introduces heterogeneous and more complex non-exponential current decay (Fig. 4a vs. Fig. 5a). For situations where multiple exponential terms were required to adequately describe the decay, a weighted time constant was used for comparisons. All results are expressed as the mean ± SEM. The non-parametric Kruskal-Wallis test was used to evaluate the statistical significance of differences in the estimated parameters.

## Additional Information

**How to cite this article**: Fineberg, J. D. *et al*. Closed-state inactivation involving an internal gate in Kv4.1 channels modulates pore blockade by intracellular quaternary ammonium ions. *Sci. Rep*. **6**, 31131; doi: 10.1038/srep31131 (2016).

## Supplementary Material

Supplementary Information
