# Supplementary material for: Closed-state inactivation involving an internal gate in Kv4.1 channels modulates pore blockade by intracellular quaternary ammonium ions
Source: Sci Rep. 2016 Aug 9;6:31131. doi: 10.1038/srep31131 (PMC4977472; doi:10.1038/srep31131)
Supplement: Supplementary Information [file srep31131-s1.pdf]

Supplementary Information

Closed-state inactivation involving an internal gate in Kv4.1 channels modulates pore blockade by intracellular quaternary ammonium ions

Jeffrey D. Fineberg[1,3] Tibor G. Szanto[4], Gyorgy Panyi[4,5] and Manuel Covarrubias[1,2,3]

[1]Department of Neuroscience, [2]Vickie and Jack Farber Institute for Neuroscience and

[3]Graduate Program in Molecular Physiology & Biophysics

Sidney Kimmel Medical College at Thomas Jefferson University

Philadelphia, PA 19107

[4]Department of Biophysics & Cell Biology, Faculty of Medicine,

University of Debrecen, Hungary

[5]MTA-DE-NAP B Ion Channel Structure-Function Research Group, RCMM,

University of Debrecen, Hungary

4032 Debrecen, Hungary

Supplementary Figures

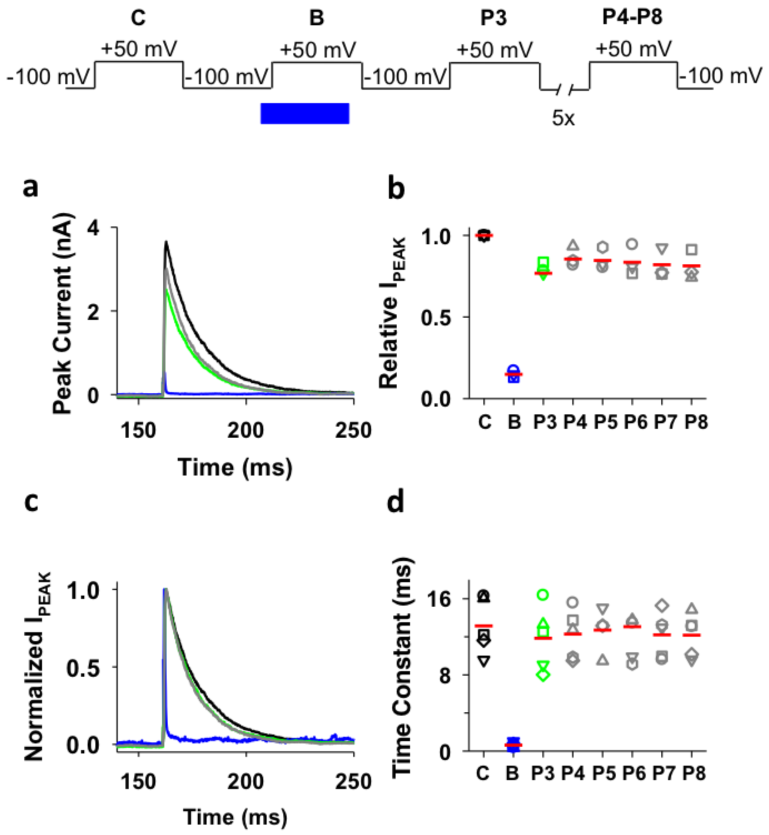

Figure 1S. Inactivation of the Shaker-IR T449K channel cannot trap internally applied bTBuA but acts as an open-channel blocker. T449K channels were transiently expressed in *tsA-201* cells (see Methods). **(a)** Outward currents evoked by a step to +50 mV from a holding voltage of -100 mV in the inside-out patch configuration. The overlaid traces depict the current profile before (P1, C=control, black), during (P2, B=block, blue) and after (P3, green, and P4-P8, grey) the exposure of the intracellular side of the channel to 100 µM bTBuA (only a 100-ms-long-segment of the currents is shown for clarity). The exposure to the QA ion began before B and was terminated after macroscopic inactivation reached steady-state (*solid* blue bar in protocol scheme above panel a). Note that no exposure was allowed during the repolarizing step that closes the channels. (**b**) Scatter plots of the normalized peak current amplitudes. Horizontal bars indicate the mean of N experiments. (**c**) Currents in panel (a) were normalized to their respective peak and shown as a function of time. Color code is the same as in panel (a). (**d**) Scatter plot of the individual time constants obtained from currents evoked by pulses C, B, and P3-P8 (see above) and the mean of the time constants (horizontal bars). Inactivation time constants were determined as described in Fig. 6 legend.

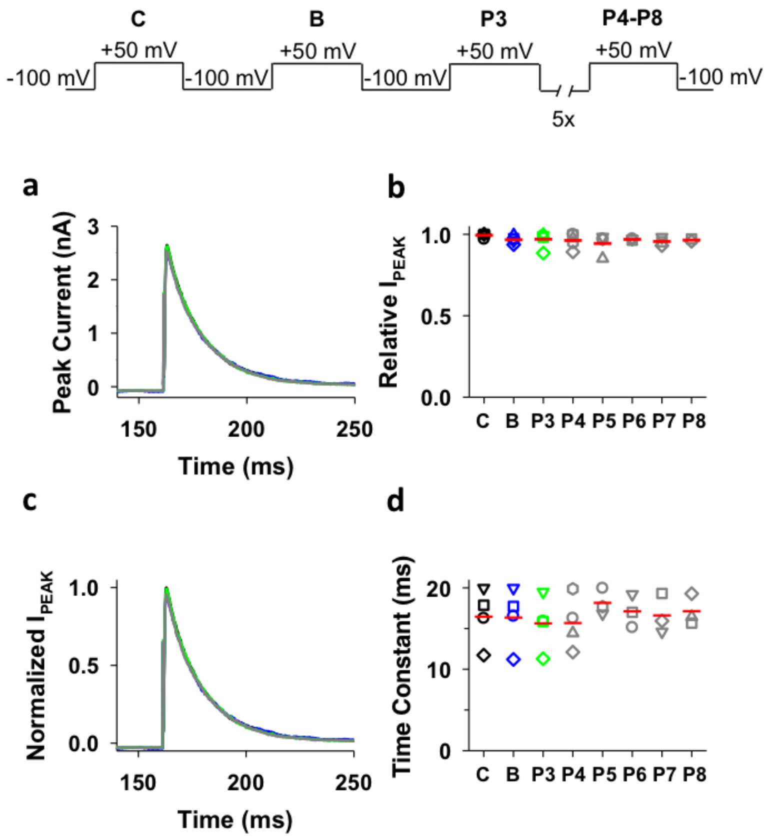

Figure 2S. Shaker-IR T449K channels are relatively stable in the absence of bTBuA. (a) Inside-out patches (*tsA-201 cells*) were repeatedly depolarized from a holding potential of −100 to +50 mV by using the pulse protocols shown in Fig. 2. The first 180-ms-segments of the 5-s-long traces are shown for clarity. **(b)** Scatter plots of the normalized peak current amplitudes. Horizontal bars indicate the mean of N experiments. **(c)** Normalized currents from panel (a). The peak currents for each pulse were determined and normalized to the peak current recorded during the first pulse (C). The peak of the normalized currents was typically ≥0.9. **(d)** Scatter plot of the individual time constants obtained from currents evoked by pulses C, B, and P3-P8 (see above) and the mean of the time constants (horizontal bars). Inactivation time constants were determined as described in Fig. 6 legend.

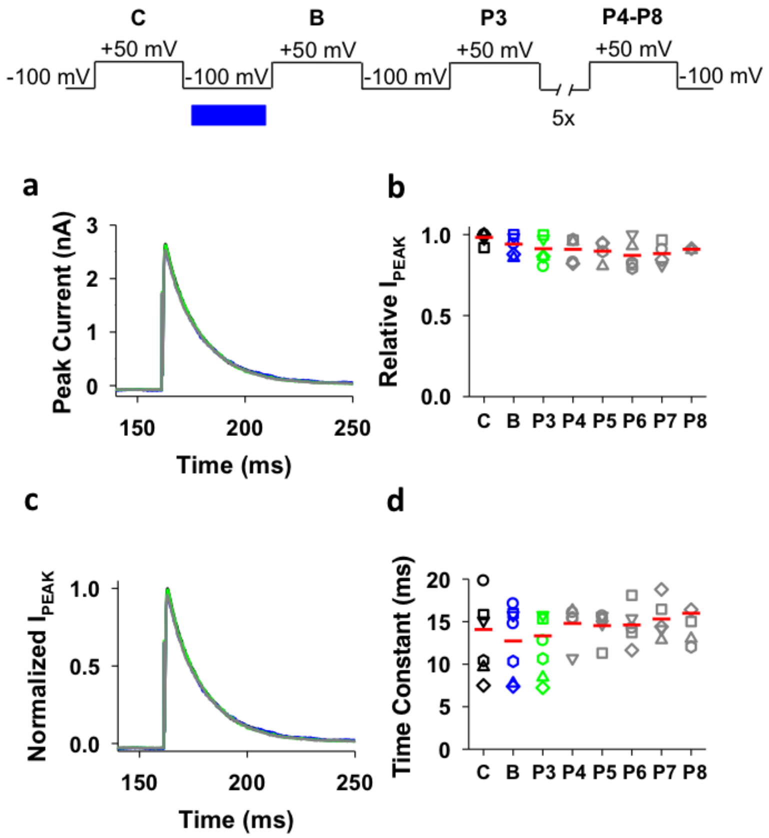

Figure 3S. Internally applied bTBuA does not block closed Shaker-IR T449K channels. Inside-out patch current recording from *tsA201* cells. **(a)** The channels were held closed at a holding potential of -100 mV, and their activity were monitored using 5-s-long activating pulses to +50 mV every 5 s. The intracellular surface of the patch was exposed to 100 µM bTBuA for 4 s during the holding potential between C and B as indicated by the *solid* blue bar. bTBuA was applied after the channels closed completely at the holding potential following C and the drug was completely washed out before B. The overlaid traces depict the current profile during C (black), B (blue) and P3 (green) (only 100-ms-long segment of the currents is shown for clarity). (**b**) Scatter plot of normalized peak currents. Horizontal lines indicate mean of N individual experiments. **(c)** Peak currents for each pulse were normalized to the peak current measured during pulse P1(C). The peak of the normalized currents was typically ≥0.9. (**d**) Scatter plot of the individual time constants obtained from currents evoked by pulses C, B, and P3-P8 (see above) and the mean of the time constants (horizontal bars). Inactivation time constants were determined as described in Fig. 6 legend.

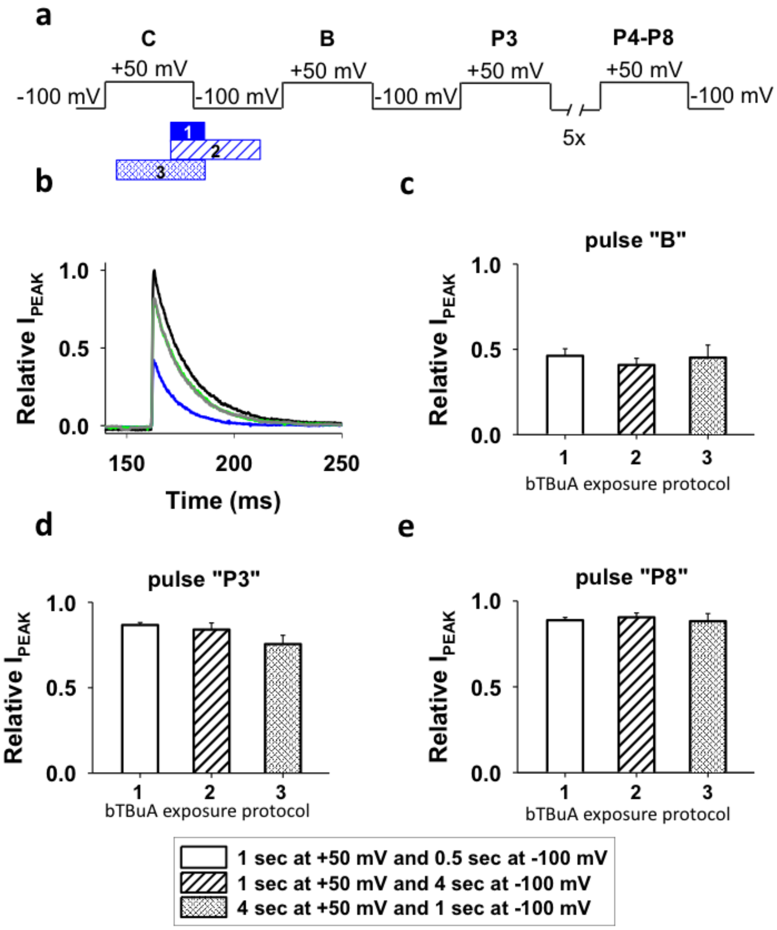

Figure 4S. Silent blockade by bTBuA in the open-inactivated and closed states does not depend on duration of exposure to the blocker. (a) Voltage-clamp protocol to probe for bTBuA ion trapping by inactivation. **(b)** Macroscopic outward currents were recorded in inside-out patches from *tsA-201* cells. The patches were repeatedly depolarized from a holding potential of −100 to +50 mV for 5 s to ensure complete inactivation. The nomenclature of the pulses in the sequence is in panel (a). The interpulse interval was 5 s and the patches were held at the holding potential between the pulses. The intracellular side of the patch was exposed to 100 μM intracellular bTBuA for 1 s at +50 mV and 500 ms at -100 mV (blue bar, 1), for 1 s at +50 mV and 4 s at -100 mV (blue hatched bar, 2) and 4 s at +50 mV and 1 s at -100 mV (blue cross-hatched bar, 3), respectively. The overlaid traces depict the currents recorded in control solution (C, black), during the first pulse following the exposure of the intracellular side of inactivated channels to 100 μM bTBuA (B, blue) and during subsequent pulses in control solution (green, P3, and gray, P4-P8). Only 100-ms-long segment of the currents is shown for clarity. **(c)**, **(d)** and **(e)** show the normalized peak current amplitudes measured during pulse B, P3 and P8, respectively, under different timing and duration of the bTBuA exposure (see above).
